# Care Provider Behaviors That Shape Parent Identity as a “Good Parent” to Their Seriously Ill Child

**DOI:** 10.1089/pmr.2021.0005

**Published:** 2021-04-27

**Authors:** Marie L. Neumann, Meaghann S. Weaver, Blyth Lord, Lori Wiener, Pamela S. Hinds

**Affiliations:** ^1^ Division of Transplant Surgery, Department of Surgery, University of Nebraska Medical Center, Omaha, Nebraska, USA.; ^2^ Division of Pediatric Palliative Care, Department of Pediatrics, Children's Hospital and Medical Center, Omaha, Nebraska, USA.; ^3^ National Center for Ethics in Healthcare, Washington, DC, USA.; ^4^ Courageous Parents Network, Newton, Massachusetts, USA.; ^5^ Pediatric Oncology Branch, Center for Cancer Research, National Cancer Institute (NCI), National Institutes of Health (NIH), Bethesda, Maryland, USA.; ^6^ Department of Nursing Science, Professional Practice and Quality, Children's National Health System, Washington, DC, USA.; ^7^ Department of Pediatrics, the George Washington University, Washington, DC, USA.

**Keywords:** complex medical needs, family, parenting, pediatric

## Abstract

***Background:*** Parents of medically complex children hold deeply personal definitions of how to be “good parents” that guide their medical decision making and interactions with providers and are impacted by provider behaviors.

***Objective:*** This study explored whether and how these beliefs are shaped by interactions with care providers and which provider behaviors foster or impede parents' ability to achieve their “good parent” definitions.

***Methods:*** A 63-item web-based survey distributed by an online support network for parents of medically complex children. Responses to closed- and open-ended questions from 67 caregivers based in the United States and Europe were analyzed.

***Results:*** Respondents' medical decisions are driven by goals of *unselfishly doing what is best for my child* (61%) and *being my child's voice* (18%). Almost half indicated that their personal “good parent” definition was impacted by provider behaviors or interactions with physicians or nurses. Although most parents reported wanting trusted care providers to ask them about their personal “good parent” definition, only 7% had ever been directly asked by members of their care teams about this topic. Provider behaviors such as kind and caring interactions, acknowledging the parents' role in caring for the child, and truly seeing the child as more than a diagnosis were reported as fostering caregivers' ability to achieve their “good parent” beliefs.

***Conclusions:*** The findings indicate that trusted provider-initiated conversations about “good parent” beliefs would be well received and are an opportunity to improve family-centered care. Care provider behaviors deemed by parents as supportive facilitate their efforts to achieve their “good parent” beliefs.

## Introduction

Parents of children with serious illness or medically complex needs develop definitions of what it means to them to be a “good parent” to their child.^1^ Prominent themes of this definition have been identified as including informed decision making, being present for the child, ensuring the child always knows they are loved, teaching the child to make good decisions, and advocating on behalf of the child.^1–4^ These definitions have been described as being distinct from one another, changing over time, providing parents with a sense of duty, and being influenced by parent–clinician interactions.^5^

The “good parent” concept functions as an identity to the parent as well as an affirming goal to work toward.^6^ Importantly, the term, originally suggested by parents of children with serious illness,^1^ does not come from a place of judgment—there is no “bad parent” in its juxtaposition.^5^ Rather, these “good parent” ideals inform parents' sense of duty and purpose, function as a guiding compass in decision making, and shape interactions with their child and with clinical personnel.^5^ Knowing they are being a “good parent” to their child helps parents cope during the hard phases in their child's illness trajectory.^7^ Bereaved parents' recognition of having reached their personal “good parent” definition during their child's life may provide them with a sense of peace after their child's passing.^3^,^8^

Incorporation of the “good parent” concept in clinical practice presents a unique opportunity to enhance family-centered care and the support offered to parents with medically complex, seriously ill, or terminally ill children.^1^,^2^,^9^ Parents' “good parent” beliefs are closely connected to the priorities they set for their child's medical care, thereby shaping the medical decisions they make for their child and the interactions they have with clinical and nursing staff.^4^ For care providers from all disciplines, understanding parental perspectives on how they aim to be “good parents” to their medically complex child provides valuable insight into parents' motivations and behaviors and offers opportunities for support.^5^,^10–12^

We solicited parental insights into how their “good parent” definition shapes their medical decision making and interactions with care providers using an online survey tool utilizing both closed- and open-ended questions. As part of the survey, we asked parents what behaviors on the part of medical professionals foster or impede their ability to achieve their personal “good parent” definition.

## Materials and Methods

The Institutional Review Board deemed the survey protocol as exempt from full review. Respondents provided informed consent before their participation in the study.

The survey was designed by an interdisciplinary study team that included two parents of medically complex children according to the Tailored Method of Survey Design.^13^ It was piloted with 20 parents who are bereaved or currently parenting children with complex medical needs. As per parent feedback, the survey was revised to include an introduction indicating the term “good parent” did not mean “perfect parent” and did not have a hidden “bad parent” at its opposite, but instead inclusively honored the good intentions of all parents and celebrated diversity in parenting approaches. The final survey also included positive parenting quotations embedded within the “advance survey” button for a less sterile survey experience. The survey was independently reviewed, piloted, revised, and repiloted.

The final *Good Parents, Courageous Parents* survey^14^ consisted of 63 open- and closed-ended questions. Data from eight questions specifically addressing a study subaim to explore parental communication and interactions with medical team members regarding the “good parent” concept are presented in this article.

This cross-sectional web-based survey was distributed by the courageous parents network (CPN) (https://courageousparentsnetwork.org/about/), a United States-based nonprofit, 501(c)(3) organization created by and for parents of seriously ill children and by the providers who care for them, with oversight from professional advisory boards. Utilization of this network provided access to a catchment cohort of parents of children with chronic, complex, or critical illness. Survey responses represent a convenience sample of parents within CPN who are either bereaved or currently parenting children with chronic or critical conditions. Invitations to participate in the survey and two reminders were sent through email and social media (through Facebook and parent blog) by CPN. The survey was open from June to August 2020.

A RedCap© questionnaire format was utilized for secure online data collection. The quantitative analyses were descriptive and univariate in nature with counts utilized for categorical variable responses. For missing responses due to skip patterns in the survey, the number of responders was used as the denominator. Frequencies and percentages are presented.

Parent responses to open-ended questions underwent semantic content analysis by the study team through MAXQDA (VERBI Software, 2020). A primary coder created a codebook and analyzed each response thematically by question (M.L.N.). A secondary coder reviewed these response themes for classification (M.S.W.). Inter-rater reliability was notably >95% between coders. Differences were resolved through team communication to reach shared consensus (M.L.N., M.S.W., and P.S.H.).

We use the terms “care provider” and “provider” interchangeably to encompass all medical professionals who interface with the family and represent the medical community. In this article, these terms are meant to include physicians, advance practice practitioners, nurses, case managers, respiratory therapists, physical and occupational therapists, and other clinical staff. We realize that the labeling of physicians as providers has been criticized^15^ and utilize the term with reluctance solely for the purpose of consistency and clarity.

## Results

Survey respondents included 60 biological mothers, 5 fathers, and 2 foster parents or legal guardians of sons (52%) and daughters (48%).^14^ The average age for living children was 7.6 years (mean duration of illness was 6.7 years). Eleven bereaved parents participated, with their children having lived a mean lifespan of six years. All but two respondents were living in the United States. Respondent demographics are provided in Table 1.

**Table 1.** Respondent Demographics Table

Category	Response	%
Relationship with child	Mother	89.55
	Father	7.46
	Foster parent or guardian	2.98
Child status	Alive	83.82
	Deceased	16.18
Child gender	Male	52.08
	Female	47.92
Parenting role	Single parent	12.5
	Coparent	87.5
Parent country of residence	United States	97.01
	United Kingdom	1.49
	Germany	1.49
Marital status	Married	87.5
	Divorced	7.5
	Single	5
Parent race/ethnicity	Asian	5
	Black/African	2.5
	Caucasian	90
	Other	2.5
Parent's highest education level	Some college	20
	Completed college	37.5
	Graduate school	42.5

Pediatric diagnoses included metabolic and genetic (41%), neurologic (22%), oncologic (15%), pulmonary (15%), and gastrointestinal (7%). Two-thirds of parents indicated active involvement of six or more medical subspecialists in their child's care. Children spent an average of 21.5 days in an inpatient hospital setting in the past year. Thirty-nine percent of families had current palliative care involvement, 24% had prior palliative care involvement, and 37% had never had palliative care included in their child's care. Fifteen percent of families had current or past hospice team involvement.

Findings related to respondents' “good parent” definition, its development over time, and caregivers' sense of living up to their definitions are described in detail elsewhere.^14^ A total of eight survey questions were analyzed to investigate the impact of provider behaviors on parents' “good parent” beliefs (Table 2).

**Table 2.** Questions Included in Analysis

Survey question and corresponding answer options		*n* (%) of responses
Closed-ended	1. Has anyone on your child's health care team ever asked you about what it means to you to “be a good parent” to your child?	*n* = 47
	Yes	3 (6)
	No	44 (94)
	2. Have you ever discussed your sense of what it means to “be a good parent” with your child's health care team?	*n* = 47
	Yes	13 (28)
	No	34 (72)
	3. What has impacted your definition of “being a good parent”? (select all that apply)	*n* = 45
	Clinician behaviors	16 (34)
	Changes in child's health	32 (68)
	Awareness of child's prognosis	37 (79)
	Spiritual considerations	21 (45)
	Nurse	10 (21)
	Physician	12 (26)
	Family	27 (57)
	Other	13 (28)
	4. Would you want health care teams to explore with you what it means to “be a good parent” to your child?	*n* = 40
	Yes	6 (16)
	No	4 (10)
	Only if they know me and my child well	26 (65)
	Unsure	4 (10)
Open-ended	5. What behaviors from medical staff foster your ability to work toward your personal definition of “being a good parent”?	*n* = 42
	6. What behaviors from medical staff impede your sense of ability to work toward your personal definition of “being a good parent”?	*n* = 34
	7. In what ways does your definition of “being a good parent” to your child influence medical decision making?	*n* = 40
	8. In what ways does your definition of “being a good parent” to your child influence the way you interact with medical teams?	*n* = 38

### Provider–parent conversations about good parent beliefs

Only 6.4% of respondents had ever been asked by a care provider about what it means to them to be a “good parent” to their child. Although 60% of respondents indicated discussing their “good parent” beliefs with family and friends, only 28% had these discussions with their providers. Eighty percent of parents reported they would want a care provider to explore with them what it means to them to be a “good parent” to their individual child. Importantly, within that affirmative response, 65% of parents depicted receptivity only *if* the team member knew the parent and child well.

### “Good parent” beliefs and medical decision making

When asked to describe how their “good parent” definition influences the medical decisions they make for their child, parent responses centered around two common themes: *doing what is best for my child* (61% of responses) and *being the voice for my child* (18% of responses). Within the first theme, respondents mentioned unselfishly putting the well-being of their child first, even if doing so is not easy for the family. For many parents, this took the shape of comfort or quality of life considerations:

“I look at the big picture, what will life look like after this procedure? Will it improve things or allow us to maintain our normal? Will it cause him to live in pain?”

“Being a good parent means doing what's best for my child, even if it is difficult/inconvenient/costly. Therefore, when our medical team indicated that we should travel across the country to explore a special surgery procedure, we did so.”

“The goal was to keep my son comfortable. Period. He deserved that. I fought through many suggestions of unnecessary interventions for him to keep this goal.”

Responses falling into the second theme highlight the importance of the parents amplifying the voice of their child in the making of medical decisions:

“For me, making medical decisions as a good parent means that I will consider all the aspects of her, not just the medical impact. Knowing who she is and what she loves means that I make decisions that allow for those things as much as possible, even if it means making harder medical decisions.”

“The first question I asked is what does my son want. Being a good parent means giving his voice the priority.”

### “Good parent” beliefs and interactions with care providers

When asked to describe how their “good parent” definition influences interactions with care providers, 69% of respondents mentioned becoming advocates for their child in interactions with their medical team, with some parents recognizing they had become more informed and courageous to advocate for their child:

“We are advocates for [our child's] comfort and feel the weight of mediating with our medical team to always make sure it is the highest priority”

“We regularly have to ask the medical teams to ‘slow down.’ It's in their nature to do more tests and more treatment. In many cases, after some serious consideration, we all reach the determination that additional tests won't change the course or treatment, no matter the result. But we're always trying to minimize the trauma of a life-limiting illness, and that often means questioning the directives of our son's medical team.”

### Impact of care provider behaviors on parent's ability to reach “good parent” definition

On a multiple-response survey question asking respondents what has impacted their definition of being a “good parent,” 34% of parents indicated clinician behaviors and interactions with physicians (25%) and nurses (21%) as shaping their good parent beliefs. In open-ended responses, parents reflected on specific provider behaviors that foster (Table 3) or impede (Table 4) their ability to reach their “good parent” definitions.

**Table 3.** Care Provider Behaviors That Foster Parents' Ability to Reach Their “Good Parent” Definition

**Theme 1: Kind and caring communication with parents (25 responses [59.5%])**		
**Subtheme 1**	**Subtheme 2**	**Subtheme 3**
**Talking openly, honestly, and clearly**	**Providing encouragement and reassurance**	**Truly listening to parents**
12 responses (28.6%)	12 responses (28.6%)	9 responses (21.4%)
Care providers provide clear explanations in ways that the parent can understand, educate the parents, and make communication easy and straightforward	Providers remind parents they are doing a good job caring for their child and provide overall reassurance and support	Care providers are fully present in conversations, not rushing, and truly listening to parents
“Their ability to be direct and honest even in the face of bad news and outcomes” “Giving detailed information about diagnosis, treatment, prognosis and palliative care options”	“[Tell] me it is okay… [that] things will turn out good” “Stick with it”. “You're doing a great job” “Their affirmations that I was doing the best I could, and acknowledging what I was trying to do for him.”	“[Being] fully present in conversations with [my child], and with me and as a result we both feel very safe and supported by them.” “We have doctors that listen to me and my input on her health and wellbeing.”
**Theme 2: Acknowledgment of parents' role in caring for the child (14 responses [33.3%])**		
**Subtheme 1**		**Subtheme 2**
**Including parent as valuable team member**		**Trusting parent judgment**
8 responses (19%)		6 responses (14.3%)
Providers honor parents' expertise in their child's well-being, treat parents as partners in decision making, include parents in discussions		Providers express confidence in parents' values as part of medical decisions, empower parents to advocate for their child
“Including me in discussions, avoiding assumptions about my son or our family” “being treated as a teammate” “honoring our expertise” “Confidence in my knowledge of my own child vs having to push my concerns”		“[Our doctor] trusts our judgment (like when we knew she had a bladder infection or was getting pneumonia) and calls in appropriate medicine” “being trusting of our intuition”
**Theme 3: Truly seeing the child (12 responses [28.6%])**		
**Subtheme 1**		**Subtheme 2**
**Seeing the whole child**		**Recognizing the importance of goals of care and quality of life**
7 responses (16.7%)		6 responses (14.3%)
Care providers directly connect with the child, personally acknowledging and regarding the child for more than their medical condition		Providers inquire about goals of care, help parents sort out priorities, act in the child's best interest, and are aware of quality-of-life goals
“I am grateful that there are all sort of amazing doctors and nurses & specialists, who truly love my child” “Any discussion of love, what was important to our family and what our daughter enjoyed doing.” “When they rely on my knowledge of her as a person, not just her medical conditions.”		“All his specialists being on board and supporting his ‘quality of life’.” “We have a great pulmonologist that also encourages us to focus on his experience of life.” “Not doing things that are convenient for care givers but instead what is optimal for my child”`

Note: Data analysis based on survey responses (*n* = 42) to the following open-ended question: “What behaviors from medical staff foster your ability to work toward your personal definition of “being a good parent”?”

**Table 4.** Care Provider Behaviors That Impede Parents' Ability to Reach Their “Good Parent” Definition

**Theme 1: Poor communication with parents (18 responses [52.9%])**	
**Subtheme 1**	**Subtheme 2**
**Not engaging with parents from a place of openness and empathy**	**Not listening to parents**
12 responses (28.6%)	7 responses (20.6%)
Providers are condescending, judgmental, ask the same questions multiple times as if to question parental response, or lack empathy	Parents describe care providers not truly hearing them or their child
“If the medical staff is condescending or talks down to me, I vacillate between being insulted and feeling somewhat inadequate.” “It is difficult when staff don't ask about our home experiences or make assumptions about how we ‘should’ behave, especially in a hospital setting” “Talking about his medical condition in front of him as if he is not there, or not able to fully understand him and not asking his input.” “Their reluctance to get into the emotional aspects of care for my child and our family.”	“not listening to how the parents and the child—a teenager—wanted to be treated and their wishes respected” “Medical staff work so hard to heal, but often forget to listen” “If they're hearing you but not really listening.”
**Theme 2: Not seeing the big picture or recognizing family impact of treatment decisions (11 responses [32.3%])**	
Care providers are overly focused on quantity of days, rather than quality of life, make therapies unrealistic, are unable to understand the care involved, and see the child as a medical case, rather than a person; some parents mention fragmented care	
“When medical staff see your child as a case and not a person, it makes it difficult to know what you can do to be a good parent.” “Adding meds that simply don't help. Wanting to try procedures that will not improve his quality of life, simply because they believe giving him quantity of life is more important.” “Each specialist is working towards optimizing the part of my son that they are focusing on and rightly so but when you add the list of treatments, meds, therapies, equipment, etc. for each when you have 17 different providers giving you information that list becomes unmanageable.” “Forcing me to spend too much time and energy on managing details and deconflicting various specialties.”	
**Theme 3: Not valuing parent expertise or wishes (12 responses [28.6%])**	
Parents describe care providers ignoring or not being interested in their wishes or not taking them seriously, not including parents in the team, asking parents to justify their decisions, or not trusting parent observations.	
“When I'm not taken seriously as a parent as part of my child's medical team.” “Making decisions or narrowing options without including me in the discussion.” “Being asked to justify our decisions, pushing for interventions we don't want OR denying our requests for info about new/different interventions.” “Doubting my concerns. Not taking my concerns serious until they spiral into a bigger issue.” “Doctors discounting mothers, medical staff treating me like I am stupid”	

Note: Data analysis based on survey responses (*n* = 34) to the following open-ended question: “What behaviors from medical staff impede your ability to work toward your personal definition of “being a good parent”?”

Within these responses, communication emerged as the most common theme. Most respondents (60%) indicated that *kind and caring communication with parents*, including talking openly, honestly, and clearly (29%), providing encouragement and reassurance (29%), and truly listening (21%), help them to be the best possible parent they can be to their child. *Poor communication,* including not engaging with parents from a place of openness and empathy (32%) and not listening (19%), was described as impediments by 53% of respondents.

Many parents (33%) also described providers' *acknowledgment of the parents' role and expertise in caring for their child* as fostering their ability to work toward their own personal “good parent” definition. Respondents mentioned inclusion of the parent as a valuable team member (19%) and trusting parent judgment (14%) as cultivating their perception of themselves as a “good parent.” Conversely, care providers not recognizing parents' expertise and wishes in caring for their child were seen by some respondents (24%) as an impediment.

A third theme emerging from the open-ended responses is of care providers *truly seeing their child* (29%). Respondents described care providers seeing beyond the child's medical condition (17%) and interacting and connecting emotionally with the child (14%) as fostering their ability to be a “good parent.” On the other end of the spectrum, providers who do not appreciate the “big picture” implications of treatment for quality of life of the child or the family were seen by many respondents (32%) as impeding their ability to be “good parents.”

## Discussion

Previous research indicates that parents of medically complex, seriously, or terminally ill children develop a deeply personal definition of what it means to be a “good parent” to their child.^1–12^ This definition is at once an identity and a goal and functions as a guiding compass for parents as they make medical decisions for their child, both small and large.^16^

Respondents reported talking about their “good parent” beliefs with friends and loved ones as part of their processing of parental roles; most respondents indicated also wanting to explore these beliefs with the care provider(s) they know well. However, only a very small subset had ever been directly asked about what being a “good parent” to their medically complex child means to them. Whether or not providers initiate discussions on parents' “good parent” beliefs, these directional virtues are often (consciously or unconsciously) informing decision making and guiding interactions. Provider-initiated conversations may serve as an invitation to name, describe, and foster shared awareness of an existing or evolving concept.

This finding of a disconnect between parents' desire to discuss their “good parent” beliefs with trusted care providers and those conversations actually being initiated by providers is novel. It is also startling, particularly because respondents recognized that interactions with providers shape how they define their roles as “good parents” to their children, and whether they see themselves as reaching their “good parent” intentions.

Parents have previously identified the role for their child's clinicians to provide validation and reinforcement of their good parenting beliefs and empowering them as important advocates for their child.^11^ Together, these results suggest that there is great opportunity within provider–parent interactions to impact parents' sense of direction as they aim to be the best possible parent they can be to their medically complex child, and to bolster and acknowledge them in their intentions.^9^

Respondents indicated that their internal definition of a “good parent” guides the medical decisions they make for their child, as has been reported previously.^2,4^ Specifically, parents orient themselves along their “good parent” intention of unselfishly prioritizing their child's best interest; for many respondents, their child's quality of life plays a central role in their selfless decision making. Furthermore, respondents think of themselves as giving voice to their children—not just regarding their medical needs but also for their child's entire being. When faced with making medical decisions, respondents seem to return to a central theme: how will this affect my child's future and their ability to live their life to the fullest, whatever that may mean?

In cases wherein parents felt that a suggested intervention would not ultimately result in meaningful improvements to their child's ability to make the most out of their life, parent respondents depicted a sense of obligation to utilize their intimate familiarity with their child and personal “good parent” definition to advocate for the care priorities they believed best honored their child's needs. Some respondents described being assertive with their medical teams, actively negotiating with care providers or refusing what they deem to be unnecessary procedures.

For parents whose absolute priority it is to make unselfish informed medical decisions with the wishes of their child honored and respected, this is a difficult and sometimes adversarial situation to negotiate. It also presents a meaningful opportunity on the part of the provider to engage with parents on their “good parent” priorities and beliefs and offer individualized support.

**FIG. 1.**
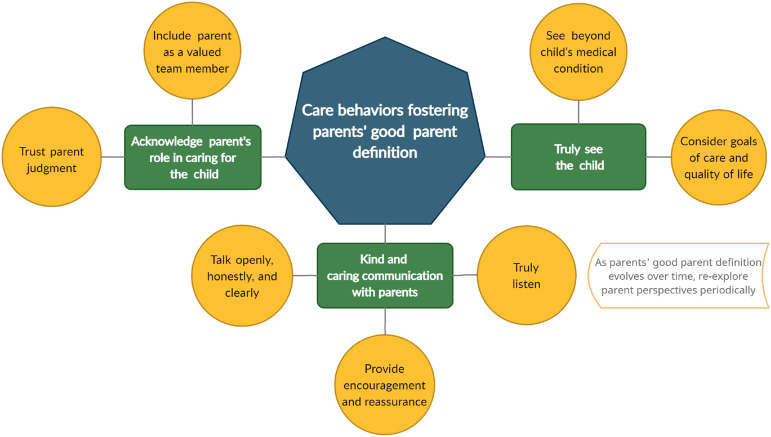
Care provider behaviors that foster parents' “good parent” identity. Based on a total of 42 responses to the open-ended survey question: “What behaviors from medical staff foster your ability to work toward your personal definition of ‘being a good parent’?”

Survey respondents offered specific ideas on how their personal “good parent” beliefs can be supported by providers, which are summarized in Figure 1. Most prominently, parents highlighted the importance of kind and caring interactions with care providers. Such interactions include clear and honest communication to inform and educate the parents, but also the provision of encouragement and reassurance and a true hearing of parent concerns. Hence, these data speak of parents' need to be truly listened to and communicated with openly and clearly about their child's medical condition, and to be reassured that, at the end of the day, they are doing good for and by their child.

Consistent with prior studies,^9,11^ survey respondents indicated that a recognition of the parent as a vital member of the medical team helps to solidify their “good parent” beliefs. For the parents who have spent years caring for their child with complex medical needs, and who have acquired a level of expertise in their child's condition and in their child's signs of well-being or illness, being discounted or not included in discussions by their medical team is disheartening. Instead, parents want to be “treated as a teammate,” have their expertise honored, and feel that care providers have trust and confidence in their judgment.

Lastly, many parents described clinicians who see the child as a medical case rather than a person, who are overly focused on quantity, rather than quality, of life, and who are unable to understand the child's experiential perspective as impeding their ability to be the “good parent” they want to be. Respondents highlighted the importance of providers seeing their child for who they are beyond their medical condition and recognizing the importance of goals of care and quality of life. When care providers can see beyond a child's diagnosis and connect with the child on an emotional level as well as prioritize quality of life and goals of care, they align themselves more closely with the “good parent” intentions of their patient's parents.

A notable strength of this research is the explicit inclusion of two parents of medically complex children in the research team and the solicitation of feedback from a group of parents in the development of the survey.

Limitations are an inability to determine a response rate as the number of parents receiving the invitation to participate through social media could not be calculated, the risk of a social-desirability response bias, a potential selection bias resulting from our recruitment of members of a parental support group, and a relatively small sample size. The study is further limited by a lack of diversity with the sample primarily consisting of educated white coparenting females from Christian tradition. The “good parent” concept is notably influenced by socioeconomic status, gender, familial, cultural, and social determinants of health. Further study on this topic in diverse communities is most certainly warranted.^4,5^

## Conclusion

Parents of medically complex, seriously ill, or terminally ill children hold a set of “good parent” beliefs that function as a guiding compass, offering orientation in an often-spinning world of clinic visits, hospital admissions, and medical encounters. We find that parents want to discuss their “good parent” beliefs with trusted care providers. Yet, providers only very rarely initiate these conversations. Care providers' compassionate and curious exploration with parents of their “good parent” beliefs may foster a sense of shared journey and even a clarifying roadmap for directions of care.

## Abbreviation Used

CPN: Courageous Parents Network
